# Autoimmune Thyroid Disorders: The Mediterranean Diet as a Protective Choice

**DOI:** 10.3390/nu15183953

**Published:** 2023-09-12

**Authors:** Rosaria Maddalena Ruggeri, Maria Cristina Barbalace, Laura Croce, Marco Malaguti, Alfredo Campennì, Mario Rotondi, Salvatore Cannavò, Silvana Hrelia

**Affiliations:** 1 Department of Human Pathology of Adulthood and Childhood DETEV “G. Barresi”, Endocrinology Unit, University of Messina, 98125 Messina, Italy; cannavos@unime.it; 2 Department for Life Quality Studies, Alma Mater Studiorum, University of Bologna, 40126 Bologna, Italy; maria.barbalace2@unibo.it (M.C.B.); silvana.hrelia@unibo.it (S.H.); 3 Department of Internal Medicine and Therapeutics, Unit of Endocrinology and Metabolism, Laboratory for Endocrine Disruptors, Istituti Clinici Scientifici Maugeri, Istituto di Ricovero e Cura a Carattere Scientifico (IRCCS) University of Pavia, 27100 Pavia, Italy; laura.croce@icsmaugeri.it (L.C.); mario.rotondi@icsmaugeri.it (M.R.); 4 Department of Biomedical and Dental Sciences and Morpho-Functional Imaging, Unit of Nuclear Medicine, University of Messina, 98125 Messina, Italy; acampenni@unime.it

**Keywords:** autoimmune thyroid diseases, Hashimoto’s thyroiditis, Mediterranean diet, Western diet, vegetarianism, oxidative stress

## Abstract

Autoimmune thyroid diseases are on the rise worldwide, and such a rapid increase is mainly driven by environmental factors related to changed lifestyles in “modern” societies. In this context, diet seems to play a crucial role. An unhealthy high-energy diet, rich in animal fat and proteins, salt and refined sugars (the so-called “Western diet”) negatively influences the risk of autoimmunity by altering the immune balance and the gut microbiota composition, enhancing oxidative stress and promoting inflammation. In contrast, the Mediterranean diet represents a unique model of healthy eating, characterized by a high intake of food from vegetable sources, a low consumption of saturated fats in favor of unsaturated fats (mainly, olive oil), a moderate consumption of fish (typically, the small oily fishes) and dairy products, as well as a moderate consumption of wine at meals, and a low intake of meat. Thanks to its nutritional components, the Mediterranean Diet positively influences immune system function, gut microbiota composition, and redox homeostasis, exerting anti-oxidants, anti-inflammatory, and immunomodulatory effects. The present review was aimed at exploring the existing knowledge on the correlations between dietary habits and thyroid autoimmunity, to evaluate the role of the Mediterranean diet as a protective model.

## 1. Introduction

Autoimmune thyroid diseases (AITDs) include a wide spectrum of disorders, ranging from hyperthyroidism and goiter (Graves’ disease, GD) to hypothyroidism and glandular hypo-atrophia (Hashimoto’s thyroiditis, HT) [1,2]. They are the result of a failure of immunological tolerance to self-antigens and consequent activation of immune responses against thyroid tissue. AITDs are multifactorial diseases, deriving from the interaction between genetic and environmental factors, which trigger the development and further progression of AITDs in genetically susceptible individuals [1,2,3,4]. GD is an uncommon disease, and its prevalence/incidence has remained stable over the years [5,6], whereas autoimmune thyroiditis (namely, HT), is very common, affecting about 3–5% of the general population, and its incidence has been increasing [7,8,9]. In recent decades, large epidemiological studies have reported increased prevalence and incidence of several autoimmune disorders, including rheumatic (systemic lupus erythematosus, rheumatoid arthritis, to mention a few), neurological (multiple sclerosis, myasthenia gravis), gastro-enteric (inflammatory bowel diseases, celiac disease) and endocrine (such as autoimmune thyroiditis, type 1 diabetes) diseases, mostly in developed countries of the West and North compared to developing countries of the East and South [10,11,12].

Such an increase clearly correlates with improved hygiene and health standards, as well as with socioeconomic status, suggesting that environmental factors are driving these geo-epidemiologic changes [1,10,11,12]. In particular, interest has been increasing in the “modern” lifestyle of industrialized, “westernized” societies, and to the potential environmental triggers that characterize it, including, for instance, reduced microbial exposures and improved hygiene, pollutants, psychological stress overload, deficiencies or excess of nutrients (notably, vitamin D, iodine, and selenium, to mention a few), whose role in autoimmunity is intensely debated [12,13,14,15,16,17,18,19,20]. In particular, in developed countries, excess calorie intake and frequent consumption of “unhealthy food”, associated with a sedentary lifestyle, has led to a rise in the prevalence of obesity, which in turn predisposes individuals to several chronic non-communicable diseases, including inflammatory and immune-mediated disorders [19,20,21,22]. For this reason, interest in nutritional patterns has grown in recent years and our understanding of the link between diet and thyroid function is rapidly expanding.

This narrative review was aimed at exploring the existing knowledge on the correlations between dietary habits, intake of different foods, and thyroid autoimmunity.

## 2. Materials and Methods

For this narrative literature review, we carried out an extensive literature search on online databases (MEDLINE via PubMed, ISI Web of Science, and Scopus), using the key terms “autoimmune thyroid disease” and “diet”. Also the MeSH terms “Hashimoto’s thyroiditis”, “autoimmune diseases”, “hypothyroidism”, “hyperthyroidism”, were included in combination with “dietary regimens”, “Western-style diet”, “vegetarianism”, “vegan diet”, “Mediterranean diet”, and “Oxidative stress”, to ensure that the majority of relevant studies have been identified. The literature search was performed up to May 2023. Reviews, meta-analyses, and original studies reporting results on the correlation between dietary regimens or specific dietary components and thyroid autoimmunity were evaluated and included in the present review according to the following criteria: English language and publication in peer-reviewed journals. Duplicates, case reports/series, and papers written in other languages apart from English were excluded. The search strategy is summarized in Table 1.

## 3. The Western Diet: Possible Links with Autoimmunity

Several studies have focused on nutrition and dietary patterns (“too much”, “too fatty”, “too salty”, “too sweet”) as risk factors for the development of autoimmune diseases in Westernized societies, and they consistently suggested these dietary traits as risk factors for rheumatoid arthritis, psoriasis, celiac, and intestinal bowel diseases, among others [21,22,23,24,25,26].

In recent decades, in Westernized countries, a high-energy diet rich in fats (mainly saturated fats, trans fatty acids, and cholesterol) and proteins (mainly meat), high in salt and refined sugars, and low in fiber has become increasingly frequent, mostly among young age groups [20,21,22]. This dietary pattern has been termed the “Western diet” (WD), and it has been associated with chronic over-nutrition and increased risk of obesity and related morbidities [20,21,22]. A major hallmark of WD is represented by excess consumption of ultra-processed food and soft drinks [27,28,29]. These are hyper-palatable and ready-to-eat products, rich in unhealthy components typical of the WD trait, such as refined carbohydrates and saturated fat, but poor in healthy nutrients, such as fiber, vitamins, and trace elements [28,29,30]. Moreover, ultra-processed food and soft drinks have a high content of additives (flavors, colors, emulsifiers, sweeteners) and other ingredients that are usually absent in “real” food (for example, hydrogenated or de-esterified oils, hydrolyzed proteins), used during industrial food processing to make the final product more appealing and palatable [27]. As consumption of ultra-processed and fast foods increases, industrial food processing and exposure to food contaminants are expanding in parallel [23,27].

The WD habits favor a condition of chronic metabolic inflammation (the so-called “meta inflammation”) both directly due to the proinflammatory effects of their components, and indirectly through increased fat mass and overweight/obesity. This condition is characterized by excess release of inflammatory cytokines (such as IL-6, IL-17, TNF-alfa), altered immune balance (CD4+ effector vs. regulatory T cells), and increased oxidative stress [20,21,31,32,33,34,35,36], and negatively influence the risk of immune-mediated disorders, including AITDs. In particular, oxidative stress, defined as an imbalance between free radical production and removal into cells, plays a crucial role in the onset and progression of several autoimmune disorders [35], by causing oxidative modifications of tissue proteins, generating novel antigenic molecules, and by further promoting inflammation and immune dysregulation [33,34,35,36]. A correlation between increased oxidative stress and the WD has been demonstrated since low consumption of fruits and vegetables causes lack of exogenous antioxidants while excess intake of animal proteins (mainly red and processed meat) and fats has been associated with increased oxidants [37].

More recently, interest has been growing in the influence of the gut microbiome in autoimmune disorders, in relation to impaired intestinal barrier functions and pro-inflammatory status. In this context, the WD pattern, which high in processed foods, refined sugar, and industrial food additives, and low in fiber from fruit and vegetables, generates an unfavorable environment in the gut and alters intestinal barrier functions and microbiota composition, leading to dysbiosis and inflammation [38,39,40,41,42]. Furthermore, altered intestinal permeability, the so-called “leaky gut” condition, allows the passage of toxins, food antigens, bacteria, and microbial products, promoting inflammation and dysregulated immune responses and triggering autoimmunity through different mechanisms [12]. On these bases, it may be speculated that a WD dietary pattern may favor the initiation and development of autoimmune disorders, including thyroid diseases, in genetically susceptible individuals [21].

## 4. The Mediterranean Diet as a Model of Healthy Eating

In contrast to the WD model, the Mediterranean diet, which is characterized by a high intake of food from vegetable sources, represents a model of healthy eating. The Mediterranean diet (MD) has its origins in a portion of land considered unique in its kind, the Mediterranean basin, which historians call “the cradle of society”, because within its geographical borders the whole history of the ancient world took place. The American Scientist Angel Keys coined the term in the middle of the last century with the publication of his pioneering observations in the ‘Seven Country Study’, where he demonstrated a lower mortality rate from coronary heart disease in the Mediterranean area [43]. The observed effects were ascribed to the different dietary habits of populations examined, especially for the proportions of energy from saturated fatty acids, mainly from meats and butter fat, and from monounsaturated fats, mainly olive oil. In particular, MD represents a natural experiment consisting in the combination of eating habits traditionally followed by individuals in the olive-growing areas bordering the Mediterranean Sea with a high consumption of plant foods (cereals, fruits, vegetables, legumes, nuts, seeds, and olives), fish and seafood, modest amounts of cheese, dairy products, meat and meat products, and moderate ethanol intake, mainly in the form of red wine and during meals. Extra virgin olive oil is the main source of lipids, and represents a high nutritional quality food for its richness in bioactive compounds, mostly phenols [44].

The MD is more than a simple nutritional model; it is conviviality, passion for food, a way of eating, promoting the quality and safety of foods and the link with the land of origin. In a word, it is a lifestyle. In fact, the Mediterranean diet is included in the UNESCO Intangible Cultural Heritage List as the best choice for a healthy life (UNESCO). It satisfies the criteria because “The Mediterranean diet involves a set of skills, knowledge, rituals, symbols and traditions concerning crops, harvesting, fishing, animal husbandry, conservation, processing, cooking, and particularly the sharing and consumption of food” [45].

Monounsaturated fatty acids and n-3/n-6 polyunsaturated fatty acids (PUFA) positive ratio, fibers, vitamins (like vitamin E, B, C and β-carotene), and minerals (selenium, zinc, iron, iodine) are key components of the MD, and account for most of its beneficial effects [46,47,48]. However, it is important to emphasize that the positive impact on health of the eating habits in MD derives from the synergy of various nutrients and bioactive compounds present in foods which interact with each other, resulting in increasing beneficial effects, which are not possible to mimic by isolating a single component [49,50].

**Figure 1:**
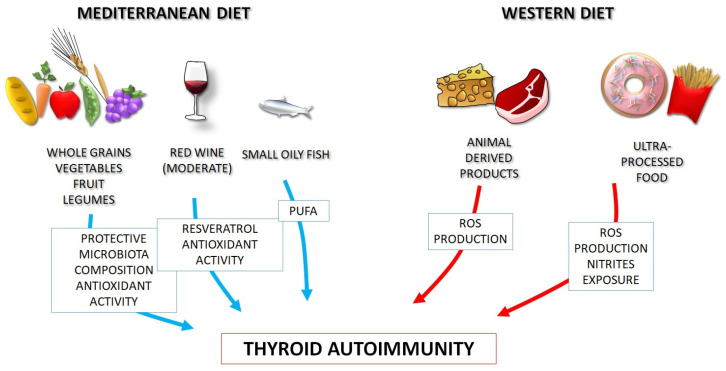
Thanks to its nutritional components, the Mediterranean diet positively influences gut microbiota, redox homeostasis and immune system function, as opposite to the Western diet, and may be proposed for prevention and management of autoimmune thyroid disorders (a “precision nutrition” approach). PUFA: polyunsaturated fatty acids. ROS: reactive oxygen species.

The health benefits of the MD are more numerous and more significant than those evidenced in the ‘Seven Country Study’, expanding beyond coronary heart disease, to the reduction in the incidence of metabolic syndrome [51,52,53,54,55,56,57,58,59]. More recently, the knowledge about the immunomodulatory, anti-oxidant, and anti-inflammatory properties of whole dietary patterns like MD has been increased, and the protective role against autoimmunity has been emerging [60,61]. On these bases, MD should represent a healthy diet model, to be recommended to patients with autoimmune disorders, including HT, as opposed to the WD (Figure 1).

## 5. Dietary Habits and Thyroid Autoimmune Diseases

### 5.1. Evidence from Clinical Studies

The association between diet and risk of developing autoimmune disorders was proposed more than 50 years ago by Trowell, who observed that autoimmune disorders, including thyroiditis, were extremely rare among isolated rural sub-Saharan populations following traditional near-vegan diets [62,63]. A similar low incidence of autoimmune disorders was reported in Asian societies whose diets were almost vegan [64,65]. In the following decades, evidence has been growing on the role of diet in the development of several autoimmune disorders, including rheumatoid arthritis, celiac and intestinal bowel diseases, type 1 diabetes, multiple sclerosis, and psoriasis [21,22,23,24,25,66,67,68,69,70,71].

Few studies, however, have evaluated the eating habits of subjects affected by thyroid disorders, and mainly in relation to thyroid function (Table 1) [72,73,74,75].

In 2015, Tonstad and coworkers investigated the association between incident and/or prevalent hyperthyroidism and dietary patterns among a large cohort of subjects belonging to the Seventh-day Adventist church, who exhibited a wide range of diets from vegan to omnivorous, with a high proportion of vegetarians. The authors observed that the prevalence of hyperthyroidism was significantly lower in subjects following a vegan diet compared to omnivores, while lacto-ovo and pesco-vegetarian diets were associated with intermediate protection [72]. Overall, this study suggested that diets excluding animal foods could be protective against thyroid dysfunction, likely autoimmune in etiology [72]. Even if the MD was not specifically studied in this case, and even if the MD does not completely exclude animal-derived products, these results can be considered relevant for our discussion. Indeed, it must be remembered that in MD, most of food intake is derived from whole grains and vegetables, with a much lower content in animal-derived food compared to the WD. Several recent studies focused on the effect of dietary patterns on thyroid function parameters. Zupo et al. investigated the possible relationship between adherence to the MD, assessed through the PREDIMED questionnaire, and thyroid function in 324 euthyroid overweight/obese subjects living in Southern Italy [73]. They found that a higher adherence to the MD was inversely related to serum levels of free T3 (fT3) and free T4 (fT4). When considering the single items of the PREDIMED questionnaire, it emerged that a higher consumption of extra-virgin olive oil was associated with lower levels of fT3 and fT4. A logistic regression model adjusted for gender, age, and BMI confirmed only the correlation between FT4 and adherence the MD, whereas no effect on serum TSH was observed [73]. The study by Liu and coworkers aimed at investigating the association between dietary inflammatory potential and thyroid function in a large population of adult males (2346 subjects), using data from the National Health and Nutrition Examination Survey [74]. The dietary inflammatory index was calculated based on the reported intake of several food groups that have anti-inflammatory potential (including fiber, vitamin C, flavonoids, garlic, several spices like rosemary and thyme) vs. pro-inflammatory ones (such as animal fat, carbohydrates, and animal protein). The results showed a positive association between dietary inflammatory index and total T4, and subjects adhering to a more pro-inflammatory diet appeared to have higher total T4 and total T3 levels, even within the normal range, while no consistent effect on fT3, fT4, or TSH could be observed. The relationship appeared to be stronger among obese subjects and those with urinary iodine concentration (UIC) levels indicating iodine deficiency [74]. The interpretation of these two recent studies performed on thyroid function parameters and dietary patterns can be cumbersome, due to the multiple factors that can influence circulating thyroid hormones, especially with normal TSH. The lack of a strong observed effect of the different dietary choices on TSH levels, and the fact that the variation in circulating T3 and T4 is of small entity and within the normal range seems to suggest that these subjects do not experience hypothyroidism, but rather small modifications in peripheral sensitivity to thyroid hormones and/or binding of thyroid hormones with transport proteins.

To our knowledge, only two recent studies specifically focused on the possible role of dietary patterns in increasing the risk of thyroid autoimmunity per se, apart from thyroid dysfunction.

In 2020, Kaličanin and coworkers evaluated the differences in food-group consumption between HT patients and healthy subjects as controls through a food frequency questionnaire (FFQ) [75]. A specific focus was put on fat consumption, in particular regarding the choice between vegetal and animal fats. The results showed that HT patients consumed more animal fat and processed meat than controls, who in turn consumed more non-processed red meat, non-alcoholic beverages, whole grains, and plant oils (Table 2). A subgroup analysis showed that HT patients on levothyroxine (LT4) therapy consumed more red meat than those who were not receiving substitutive therapy [76]. Moreover, from analysis of food consumption, it emerged that HT patients do not modify their dietary habits upon disease diagnosis, indicating that nutritional aspects were disregarded by both the patients and the physicians [75]. A more recent study by Ruggeri et al. evaluated the dietary habits of a cohort of euthyroid subjects from Southern Italy and found significant differences between HT patients and healthy subjects [37]. In particular, HT subjects consumed higher amounts of animal-derived food, including meat (both fresh and processed), dairy, and fish, and commercial sweetened products, while controls reported higher intake of vegetables, legumes, and nuts (Table 2). Of note, HT subjects and healthy controls did not differ in terms of either body weight or BMI, most of them being of normal weight. Adherence to the MD, as assessed by PREDIMED questionnaire, was lower in HT patients compared to controls, and in a multivariate logistic regression model, the PREDIMED score was an independent predictor of thyroid autoantibodies positivity, suggesting a protective role of the MD against thyroid autoimmunity [37].

### 5.2. Pathophysiological Bases of the Link between Dietary Components and Thyroid Autoimmune Diseases

**Figure 2:**
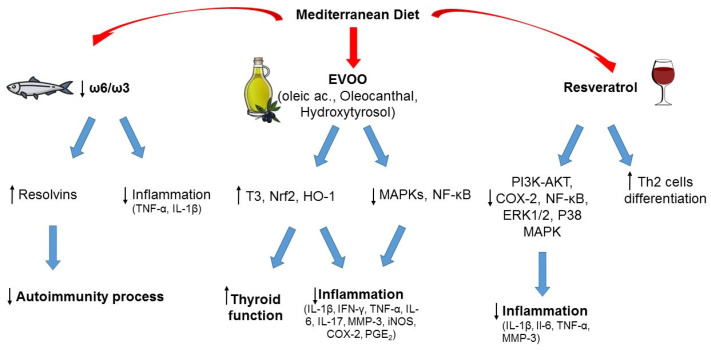
Typical foods from Mediterranean diet and their possible role in the prevention of autoimmunity and inflammation. ↑ increased; ↓ decreased.

The pathophysiological mechanisms underlying the relationship between MD, WD, and thyroid autoimmunity are not completely understood, but some hypotheses can be made, and the possible role of the various food components can be discussed, as exemplified in Figure 2.

#### 5.2.1. Animal Products

The lower consumption of animal-derived products in subjects adhering to the MD when compared to the WD appears to have a central role in protecting against thyroid autoimmunity and/or dysfunction, as suggested by pre-clinical studies [76,77,78]. Among the possible mechanisms that could be involved in this association, it could be hypothesized that high intake of animal fat could increase the risk of autoimmunity due to an increase in ROS production. Indeed, in a 2016 study by Ruggeri et al., a higher consumption of animal-derived products in patients with TH was related to a higher concentration of oxidant factors and a lower concentration of antioxidants [37]. Another reason that could explain the effect of meat consumption, especially if processed, on thyroid autoimmunity is the intake of nitrates and nitrites. Nitrites can be found in several processed foods, in particular industrial processed meat. Indeed, exposure of laboratory animals to extremely high concentrations of nitrites (~10–600 times more than the acceptable daily intake) can induce several anti-thyroid effects, including reduced levels of circulating thyroid hormones and histomorphological changes in thyroid gland. However, it should be acknowledged that no such effects have been documented in humans [79].

The role of the other main source of animal-derived food, that is fish, is more controversial in terms of effect on thyroid function and autoimmunity [37,50,80]. The MD promotes fish consumption rather than meat. Fish represents the main dietary source of omega-3 PUFAs, whose positive effects on chronic inflammatory and immune-mediated disorders are well known (see also below). On the other hand, the risk of fish contamination by environmental pollutants should be not overlooked, and a cost (contaminants) to benefits (healthy components) ratio should always be considered [50,80]. Of note, the MD is characterized by prevalent consumption of small oily fishes (the so-called “blue fish”, like anchovies, sardines, mackerels, etc.), that represent a good source of proteins and PUFAs and are associated with a low risk of contamination, whereas large, top-predator fishes (like swordfish) tend to concentrate pollutants, in particular heavy metals, persistent organic pollutants, and microplastics, due to mechanisms of bioaccumulation and biomagnification [50,80]. Several studies suggest that consumption of PUFAs, in particular omega-3, can have protective effects on the development of autoimmune thyroiditis, while heavy metal exposure was associated with an increased risk of autoimmunity [50,80]. Thus, the kind of fish consumed seems to play a role, rather than consumption of fish per se. When including fish with low content of contaminants, the MD represents a healthy nutritional model, because fish is part of a complete healthy dietary plan, and patients would benefit from both fish intake and other micronutrients [50].

#### 5.2.2. Monounsaturated and Polyunsaturated Fatty Acids (Omega-3)

Linoleic acid and alpha-linolenic acid are essential fatty acids, as their intake totally depends on foods because they cannot be synthesized in the human body, and represent the precursors of ω-6 and ω-3 PUFA families. Besides alpha-linolenic acid, the other two main ω-3 PUFAs, directly derived from it, are eicosapentaenoic acid and docosahexaenoic acid. The main difference between the two classes of PUFA is the position of the double bonds on the carbon chain (the ω-carbon): ω-6 PUFAs have the first double bond at the sixth carbon, while ω-3 PUFAs have it at the third carbon starting from the methyl end of the carbon chain. ω-6 PUFAs can be found in vegetable oils (soybean, corn, sunflower oils) in the form of linoleic acid, and animal products, whereas green leafy vegetables are a major source of ω-3 PUFAs in the form of alpha-linolenic acid. Fish and fish oil provide a good supply of eicosapentaenoic and docosahexaenoic acid [81]. Both ω-6 and ω-3 PUFAs are precursors of inflammatory mediators but with opposite effects: ω-3 PUFAs reduce inflammation whereas ω-6 PUFAs tend to promote inflammation. For this reason, a balanced ω-6/ω-3 ratio is important to support the anti-inflammatory profile of ω-3 PUFAs, and a varied diet that includes balanced amounts of each PUFA class has prevalent anti-inflammatory effects. Different from the WD, which is characterized by an excessive ω-6 PUFA intake associated with a higher production of inflammatory cytokines [46,82], the MD ensures high consumption of ω-3-rich food promoting a better inflammatory profile [46].

Dietary ω-3 PUFAs can reduce inflammation through different mechanisms, for example by lowering the synthesis of cytokines (such as TNF-α, IL-1β), prostaglandins, and leukotrienes; by controlling leucocyte chemotaxis, adhesion molecule expression, and leucocyte–endothelial adhesive interactions [48,61]. In recentyears, new classes of lipid mediators have emerged as key players in the resolution of inflammatory process. They are endogenously produced during inflammation from PUFAs and exert potent anti-inflammatory actions, serving as specialized pro-resolving lipid mediators [83]. Bioactive metabolites from ω-3 PUFAs, such as resolvins, maresins, and protectins, demonstrated beneficial effects on global health. Among them, resolvins have a well-documented anti-inflammatory activity [84]. Increasingly, evidence has demonstrated a major role of these metabolites in the regulation of autoimmune processes [61,85].

#### 5.2.3. Extra Virgin Olive Oil (EVOO)

Among the central foods of MD is EVOO. With more than 200 different bioactive compounds detected, including phenolics, sterols, carotenoids, and triterpenic alcohols, it represents the main fat used in the MD and is a hallmark of this dietary pattern. The phenolic composition of EVOO has been associated with its strong antioxidant activity, and it is thought to be largely responsible for the pleiotropic positive effects of the MD [44,86]. Recently, interest has grown in investigating the potential advantages of EVOO consumption in the context of autoimmunity. In several experimental models of autoimmune disease, EVOO components, namely hydroxytyrosol and oleic acid, and oleocanthal, were revealed to be efficacious in counteracting oxidative and inflammatory imbalances by enhancing Nrf2/HO-1 and inhibiting of MAPKs/NF-κB signaling pathways [87,88,89,90]. In two studies, the EVOO-diet supplementation was compared with a sunflower oil-diet supplementation, and both investigations reported greater EVOO effectiveness, probably due to its phenolic composition [89,90]. Olive derivatives showed varying effects on circulating thyroid hormone levels in animal models (both euthyroid and with experimentally induced autoimmune thyroiditis), with an increase in total T3 levels as the most frequently observed one [91]. In a review by Pang et al., the analyses of the literature demonstrated that olive oil, olive leaf extracts, and solid olive residue enhanced thyroid function in both euthyroid and hypothyroid animals, and in the latter ameliorated the oxidative status [91]. Obviously, further studies are required, and these results need to be confirmed in humans. Overall, the results from these studies are encouraging and provide preliminary evidence for the positive value of an EVOO-rich diet like MD to counteract and prevent autoimmune diseases.

#### 5.2.4. Phenolic Compounds of Wine (Resveratrol)

A moderate wine intake, especially red wine, with meals, is considered another unique habit in the context of the MD, with beneficial health effects related to its phenolic composition [92]. Indeed, wine is rich in phenolic compounds derived from the grapes during the wine-making processes. Resveratrol, a stilbene localized in grape skin, is acknowledged as an anti-cancer, anti-inflammatory, neuroprotective, and antioxidant bioactive compound [93,94,95]. Recently, several experimental studies evidenced a potential protective effect of this compound in different autoimmune settings, through the regulation of antioxidants, inflammatory mediators, and Th cell subpopulation balance [96,97,98,99]. Interestingly, a moderate consumption of alcohol was associated with a reduced risk of HT and hypothyroidism in the study by Carlé et al. [100]. The positive effects derived from the consumption of wine are currently under debate, but we must focus on a moderate wine consumption during meals as usually done in the MD habits. Epidemiological and clinical studies, and observations on Mediterranean cohorts of subjects, have demonstrated that moderate wine consumption correlates with benefits to human health [101]. Our intent here is not to promote alcohol as a health element or to induce people to drink alcohol in the hope of gaining benefits, rather to emphasize the role of moderate alcohol consumption, particularly red wine, within the MD and how there are no results to support complete abstention, in order to reduce the risk of developing autoimmune diseases.

#### 5.2.5. Fibers, Vitamins, and Trace Elements

The MD also stands out for the increased consumption of several food groups that can have a positive influence on thyroid autoimmunity. First, the abundant consumption of vegetables, fruits, and legumes can influence the gut microbiota composition, due to their high content in complex carbohydrates and vegetal fibers. Several studies show that patients with autoimmune thyroid disorders display alterations in gut microbiota composition and metabolism. It is well known that gut microbiota influences the host immune system via epigenetic mechanisms, and any changes in human microbiota—the so-called dysbiosis—may alter immune homeostasis and ultimately result in the development of autoimmune disorders [41]. Although the relationship between these alterations, dietary patterns, and autoimmunity has not been completely elucidated, it could be hypothesized that a favorable microbiota composition due to MD could be protective towards the development of thyroid autoimmunity [38,39,40,41,102,103]. Moreover, the MD is particularly rich in several micronutrients that have been implicated in several protective mechanisms towards thyroid dysfunction and/or autoimmunity, although in some cases with inconclusive evidence [41].

*Iodine*

Iodine is an essential trace element required for the synthesis of thyroid hormones, and a daily intake of 150 μg of iodine is recommended for adults, whereas pregnant women need a greater iodine intake (250 μg/day) [104].

Severe iodine deficiency, defined as a median urinary iodine concentration (UIC) < 100 μg/L, causes impaired thyroid hormone synthesis and represents the major cause of hypothyroidism worldwide. Chronic iodine deficiency is associated with the development of goiter and thyroid nodules [105]. During pregnancy, it results in an increased risk of growth retardation and brain damage for the fetus, and both maternal and fetal goiter [105,106,107,108]. Universal salt iodization is recommended to eradicate iodine deficiency, and the use of iodized salt is considered safe in the general population [109]. On the other hand, excess (>300 mg/d) supplemental iodine, or high iodine-rich food (e.g., seaweeds) may be harmful to the thyroid gland [16]. This can happen from the intake of supplemental iodine besides iodized salt (for instance, weight loss products) and from some dietary sources, such as seaweed (i.e., nori, kelp, kombu, wakame) that are typical of traditional Asian cuisine and are now frequently consumed in Western countries [110,111]. Moreover, an increase in thyroid autoimmunity has been observed in iodine-deficient countries after the implementation of iodine supplementation programs, but this phenomenon was transient and did not result in an increased prevalence of thyroid dysfunction in the long run [16]. A U-shaped relationship exists between iodine intake and thyroid autoimmunity/dysfunction, so that both iodine excess and deficiency are harmful to the thyroid gland, and are specifically associated with increased prevalence/incidence of AITDs [16]. Thus, adequate iodine supplementation, defined as a population median UIC between 100 and 300 μg/L, appears to be safe, and the benefits largely outweigh the potential risks [16].

Dietary sources of iodine include seafood (seaweed, marine fish, and shellfish), milk and dairy products, eggs, and poultry. Iodine can also be found in plant foods, such as cereals and grains, its content depending on the amount of iodine in the soil in which food is grown, which varies widely between different countries [112]. The intake of iodine coming only from food sources for subjects following the MD is highly variable, mainly dependent on the intake of fish [113], with some reports suggesting that the MD alone could provide an insufficient intake of iodine [114]. Nevertheless, it can be suggested that a varied and balanced MD paired with regular iodized salt consumption would allow for sufficient intake of iodine without exposing subjects to the risk of an excessive iodine intake. It should also be highlighted how vegan/vegetarian diets, despite sharing with the MD the benefits of plant-derived foods, do not provide enough iodine and supplementation is often needed [115,116]. For these reasons, subjects following a vegan diet may be at risk of either deficiency (lack of animal products) or excess (consumption of vegan alternatives, such as seaweed) of iodine and related thyroid complications [116]. Changing dietary habits, with vegetarian diets and whole-salt use becoming increasingly popular among the general population, has led to the “re-emergence” of iodine deficiency in industrialized countries with deleterious effect on children’s health [117].

*Selenium*

Selenium, a micronutrient contained in yeast and unprocessed cereals, has been long implicated in thyroid function and autoimmunity due to its antioxidant properties, and its involvement in immune function as well as in thyroid hormone synthesis and metabolism [118,119,120]. Indeed, selenium is an essential cofactor for several selenoproteins, including the antioxidant enzymes glutathione peroxidases (GPx) and thioredoxin reductases (TrxR), iodothyronine deiodinases (DIOs), and selenoprotein P, all of which are essential for proper thyroid function [120]. While even small amounts of selenium are sufficient for DIOs activity, a reduced selenium intake affects GPx expression and function, resulting in impaired antioxidant activity in thyrocytes [120]. For these reasons, selenium deficiency has been associated with thyroid dysfunction (mainly, in combination with ID) and increased risk of thyroid autoimmunity [121,122].

Although experimental studies have provided evidence in favor of a protective role of selenium against oxidative damage and apoptosis in thyrocytes [17,123,124], clinical trials aimed at evaluating the effects of selenium supplementation on AITDs provided sometimes conflicting and largely inconclusive results [125,126,127], suggesting that its impact on clinical outcomes is rather low, and selenium should not be routinely supplemented in individuals who are not deficient [128]. Nevertheless, an appropriate dietary intake of selenium is safe and useful to human health, the recommended need being 55 μg/day for healthy adults, 0.2 μg/kg/day for children, and 65–75 μg/day for pregnant and breastfeeding subjects [129].

Selenium is present in foods in two forms: inorganic (selenate and selenite) and organic (selenomethionine and selenocysteine) [130]. Typically, the main food sources of selenium are animal products, like seafoods, organ meats, eggs, dairy products (even fat-free or low-fat ones), and nuts. Plant-derived foods like grains and some fruits and vegetables also contain selenium, with amounts varying widely in relation to the selenium content in the soil and several other factors [130]. Once again, a varied and well balanced MD model meets the nutrient requirements of selenium since it includes grains (at least half whole grains), dairy products (mainly, low-fat ones like milk, yogurt, and some kind of cheese), a variety of protein foods (mostly fish, but also poultry, eggs, legumes), and nuts [131], even if evidence from the literature is not enough do draw conclusions [132].

*Iron and Zinc*

Apart from its main function related with the transport of oxygen, iron is required for the synthesis of thyroid hormones, because the thyroid peroxidase is a heme-dependent enzyme [121,122,133]. For this reason, iron deficiency can be accompanied by a reduction in thyroid hormone synthesis and a subsequent increase in TSH levels, thereby enhancing the risk of developing thyroid disease [121,122,134]. Iron deficiency may occur as a consequence of both an inadequate dietary intake or malabsorption. Iron from food comes in two forms: heme and non-heme. Heme iron derives from hemoglobin, and it is present in animal foods, like red meat, fish, and poultry (meat, poultry, and seafood containing both heme and non-heme iron). Non-heme iron is found in plant foods, like whole grains, nuts, seeds, legumes, and leafy greens. Non-heme iron is also the form of iron added to iron-enriched and iron-fortified foods. Heme iron is better absorbed by the body than non-heme iron, and some dietary components can improve (vitamin C) or inhibit (bran fiber, phytates, and tannins) its absorption when taken in the same meal [135]. Also, iron malabsorption often occurs in patients suffering from HT due to associated autoimmune comorbidities (namely, celiac disease and atrophic gastritis) [9,121,122].

Like iron, zinc is also deeply involved in thyroid homeostasis, contributing to the synthesis and activation of thyroid hormones at several levels: by regulating deiodinases activity, TSH releasing hormone, and TSH production, as well as by modulating transcription factors involved in the synthesis of thyroid hormones [136,137]. Serum concentrations of zinc also influence serum concentrations of T3, T4, and TSH [137]. However, while specific populations (such as children with Down syndrome or patients at risk of severe malnutrition) appear to greatly benefit from zinc supplementation in terms of thyroid function, the role of zinc supplementation in the general population as a protective factor for thyroid autoimmunity has not been elucidated yet [138,139,140]. Nevertheless, an appropriate zinc nutritional status is fundamental for the maintenance of a normal thyroid function in HT patients [141,142], and an appropriate dietary intake is recommended. Dietary sources rich in zinc are meats, poultry, and seafood, but legumes and whole grains can also be good sources of this trace element, taking into account that they also contain phytates, reducing its absorption.

The MD is rich in zinc, since this micronutrient is abundant in seeds and whole-grain cereals. Moreover, animal products are not excluded in this dietary regimen, so that an appropriate intake of both zinc and iron is reached. In contrast, vegan and vegetarian diets may not provide enough iron and zinc, and the use of supplements or fortified foods may be needed [142,143].

*Vitamins*

Vitamin B12 is essential for the appropriate functioning of the immune system and its deficiency is associated with an alteration in the methylation reactions leading to an increase in the levels of homocysteine in the body, which in turn favors immune dysfunction [144,145]. All foods of animal origin are good sources of vitamin B12, including meat, fish, eggs, dairy products, and poultry. As a consequence, highly restrictive plant-based regimens, like veganism and vegetarianism, are usually associated with vitamin B12 deficiency [144]. Besides dietary intake, malabsorption should be taken into account, mainly due to atrophic gastritis, celiac disease, and inflammatory bowel diseases, all autoimmune in etiology and frequently associated with AITD [9,144]. A recent study analyzed the levels of vitamin B12 in patients with AITD and found that they were significantly lower in patients than controls (*p* < 0.0001), and were inversely correlated with TPOAb levels. Moreover, HT patients with vitamin B12 deficit exhibited significantly higher mean values of TPOAb, suggesting that the determination of vitamin B12 status could be performed in HT patients as a diagnostic test with high sensitivity and good specificity [146].

Vitamin D is a fat-soluble steroid with multiple functions in the human body. Besides the well-known role in skeletal homeostasis, vitamin D acts as an immunomodulator in both innate and adaptive immune responses [147,148,149]. Moreover, its receptor is expressed in immune cells [147] as well as in several peripheral tissues, including the anterior pituitary [150] and the thyroid gland [151], suggesting a local action of vitamin D that may be relevant in the immune response. These findings are supported by the correlation between some polymorphisms of the vitamin D receptor gene and the occurrence of several autoimmune diseases, including HT [15,152,153]. In this light, vitamin D may act as a protective agent against autoimmunity, even if its role in autoimmune thyroiditis is not completely elucidated [154]. Clinical studies demonstrated that HT patients have lower serum levels of vitamin D than healthy controls [155], and vitamin D deficiency has been correlated with an increased risk of HT and also with higher levels of autoantibodies [156,157,158]. However, it is still unclear if this association is a pathological mechanism, a cause–effect relationship, or a mere “coincidence” due to the magnitude of hypovitaminosis D in the general health population [159]. Interestingly, different investigations proved that adequate levels of vitamin D were correlated with a reduction in the levels of TPO autoantibodies, suggesting a role of vitamin D supplementation as adjuvant of therapies [155,160,161]. Nevertheless, intervention trials showed only marginal effects on the reduction of TPO-antibody titers in treated HT subjects and failed to prove an effect of vitamin D supplementation on clinical outcomes [162]. In humans, vitamin D can be obtained from dietary sources or from UV-mediated synthesis in the epidermal layer of the skin. Few foods naturally contain significant amounts of vitamin D: cod liver oil and oily fish are rich in vitamin D, while butter, cream, and egg yolk contain only small amounts, as do human and cow’s milk [163]. Recent studies demonstrated that high adherence to the MD, mainly fish and olive oil consumption, are positively associated with serum levels of vitamin D, irrespective of anthropometric and lifestyle variables, skin phototype, and season [164,165].

## 6. Role of Food Contaminants

Lastly, different dietary choices could impact on the risk of thyroid autoimmunity through different exposure to environmental pollutants. Indeed, several compounds, classified as endocrine disruptors, can influence thyroid function and increase the risk of thyroid autoimmunity [166,167,168]. Xenobiotics can trigger thyroid autoimmunity via several mechanisms. They can affect factors like intestinal permeability, microbiota composition, or hormone homeostasis, all known to interact with the immune system. They can also induce epigenetic changes (such as DNA methylation, histone modifications, non-coding RNAs) in exposed cells. Finally, chemicals can act directly on thyrocyte and/or immune cells, by binding to receptors (for instance, the aryl hydrocarbon receptor AhR) or after uptake in the cells by pinocytosis, endocytosis, or diffusion. As a consequence, chemical exposures can lead to inflammation, increased oxidative stress, altered immune function (e.g., impaired Treg function, Th1/Th2 skewing, release of proinflammatory cytokine), cell apoptosis or death, and impaired function [166,167,169]. Mechanisms of molecular mimicry are also involved [169].

**Figure 3:**
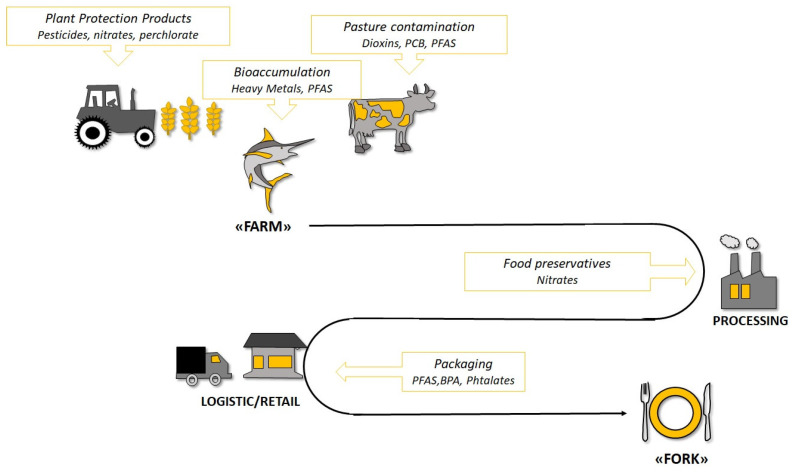
Possible sources of endocrine disruptor exposure through food consumption in the “From Farm to Food” process. PFAS: Per- and polyfluoroalkyl substances; BPA: Bisphenol A; PCB: Polychlorinated biphenyl.

Food may be contaminated at several points along the way “from farm to fork” (Figure 3). Possible sources of contamination may be: use of antibiotics in livestock feed; pollutant accumulation in fishes and crops (for instance, heavy metals, pesticides, and herbicides); anthropogenic activities such as manufacturing and food processing (additives, preservatives); contaminants adsorbed into foods from packaging materials; and deposition of contaminants during cooking [170,171]. A deep analysis of these contamination sources is far from the aim of the present review. However, their understanding allows the opportunity to briefly discuss the risk of exposure to contaminants of different dietary patterns.

It could be hypothesized that the WD characterized by increased consumption of animal-derived (meat and fish) and processed foods may be associated with increased exposure to endocrine disruptors (additive, nitrites, antibiotics, phthalates, bisphenol, polyfluoroalkyl substances or PFAS, mercury, to mention a few), compared to the MD, typically characterized by reduced intake of industrial foods and meat, and by prevalent consumption of small oily fishes with few contaminants. Moreover, the MD is characterized by a high intake of fruits and vegetables rich in flavonoids that can promote the elimination of pollutants in tissues and/or counteract their harmful effects through different mechanisms, including ROS scavenging, chelation of metals, epigenetic up-regulation of detoxifying genes (codifying for receptors like AhR, enzymes like the cytochromes). On the other hand, persistent pollutants, like PFAS, polycyclic aromatic hydrocarbons (PAHs), and PCB, accumulate in water and soil and can contaminate plant foods, in addition to those released by food packaging. The same is true for pesticides and herbicides that may contaminate crops. For these reasons, data regarding the risk of exposure to pollutants through the MD are controversial. While some authors suggest that the MD could be protective toward exposure to pollutants [172], a study by Melough et al. demonstrated that different kinds of diets, even those considered healthier, like the MD, would be equally at risk for exposure to endocrine disruptors [173]. From this study, it emerged that most endocrine-disrupting chemicals had no association with the different dietary patterns, and recommended healthy diets were not more protective against pollutant exposures than other dietary models considered unhealthy [173]. Further studies are needed to identify effective strategies to reduce dietary exposure to potentially harmful pollutants.

## 7. Limitations

The present is a narrative review focused on the hypothesis that different dietary habits may influence the onset and progression of autoimmune thyroid disorders, and the Mediterranean diet may be protective against them. We analyzed the relevant data from the literature to provide the reader with updated information on this research question. A systematic review of the literature, as well as a metaanalysis in which the results of the studies are combined statistically, could provide a more comprehensive understanding of the state of the art and detect clinically meaningful differences between dietary habits, but studies on this topic are few and heterogeneous. Clearly, further studies are needed to better understand the role of dietary habits in AITDs.

The present review cannot evaluate in depth the complex interplay between diet, microbiota, and autoimmunity. This is a relevant topic that deserves a more in-depth and extensive discussion. There are a number of detailed, well-done reviews on this topic [41,103,104].

## 8. Conclusions

Even if further studies are needed to clarify the relationships between dietary habits and AITDs, current evidence from the literature suggests that a predominantly plant-based Mediterranean diet has a potentially protective effect against thyroid autoimmunity. Reducing the intake of animal proteins and fats and increasing those from vegetable sources may represent a useful lifestyle strategy for reducing the risk for thyroid autoimmunity.

## Figures and Tables

**Table 1:** Narrative review searching strategy.

Items	Specification
Date of Search	31 May 2023
Databases and other sources searched	MEDLINE (via PubMed) and Scopus
Search terms used	First-step search: “autoimmune thyroid disease”, “Diet” Second-step search: “autoimmunity”, “Hashimoto’s thyroiditis”, “hypothyroidism”, “hyperthyroidism”, “dietary regimens”, “Mediterranean diet”, “western-style diet”, “vegetarianism”, “vegan diet”, “Oxidative stress”
Timeframe	No restrictions
Inclusion and exclusion criteria (study type, language restrictions)	Type of studies included: Clinical trials, meta-analyses, randomized controlled trials, narrative and systemic reviews Type of studies excluded: Case reports and case series, opinions Language restrictions: Abstract in English. Articles were excluded for irrelevance to the topic in question, duplicates, or for presence of original articles on the same topic that are more recent and/or with larger number of cases.
Selection process	A 2-step selection process conducted by 3 reviewers (RMR, MCB, LC) independently of each other. Articles were selected on the basis of relevance of title and abstract in the topic.

**Table 2:** Summary of studies (either clinical or experimental) evaluating the relationship between dietary habits and thyroid functional status and/or autoimmunity.

**Study**	**Dietary Habits**	**Design of the Study**	**Thyroid Effects**
Tonstad et al. [72]	Vegetarian diets vs. omnivorous diets	Observational clinical study on 65,981 subjects, members of the Seventh-day Adventist church	Lower risk of prevalent hyperthyroidism in vegan (OR = 0.49; 95% CI 0.33–0·72), lacto-ovo (OR = 0.65; 95% CI 0.53–0.81), and pesco-vegetarian (OR = 0.74; 95% CI 0.56–1.00) diets than in omnivorous diets
Zupo et al. [73]	Adherence to the MD (PREDIMED score) EVOO consumption	Observational study on a cohort of 324 euthyroid overweight/obese subjects (228 F and 96 M, aged 14–72 years)	Inverse relation with serum fT3 (*p* < 0.01) and fT4 (*p* < 0.01) levels; no effect on serum TSH
Liu et al. [74]	Dietary inflammatory potential (DIP) score	Cross-sectional study including 2346 U.S. male subjects aged ≥ 20 years (data from NHANES)	Positive association with serum TT4 (β = 0.07; *p* = 0.0044); no effect of serum fT3, fT4 or TSH
Kaličanin et al. [75]	Food group consumption frequency	Observational study including 491 HT patients and 433 controls	↑ consumption of animal fat (OR 1.55, *p* < 0.0001) and processed meat (OR 1.16, *p* = 0.0012) in HT pts. ↑ consumption of red meat (OR 0.80, *p* < 0.0001), non-alcoholic beverages (OR 0.82, *p* < 0.0001), whole grains (OR 0.82, *p* < 0.0001), and plant oil (OR 0.87, *p* < 0.0001) in controls Association of plant oil consumption with increased fT3 levels in HT patients (β = 0.07, *p* < 0.0001)
Ruggeri et al. [37]	Food group consumption frequency Adherence to the MD (PREDIMED score)	Observational study including 81 (71 F, 10 M) HT patients and 119 (102 F, 17 M) controls	↑ intake frequencies of animal foods (meat, *p* = 0.0001; fish, *p* = 0.0001; dairy products, *p* = 0.004) in HT pts ↑ intake frequencies of plant foods (legumes, *p* = 0.001; fruits and vegetables, *p* = 0.030; nuts, *p* = 0.0005) in controls Lower adherence to the Mediterranean diet in HT patients compared to controls (*p* = 0.0001) PREDIMED score was a predictor of TPOAb positivity (OR 0.192, 95% CI 0.074–0.500, *p* = 0.001)

TSH: thyroid stimulating hormone or thyrotropin; TT4: total thyroxine; fT4: free thyroxine; fT3: free triiodothyronine; TPOAb: anti-thyreoperoxidase antibodies; MD: Mediterranean diet; PREDIMED: PREvención con DIeta MEDiterránea; EVOO: extra-virgin olive oil; NHANES: National Health and Nutrition Examination Survey; ↑ increased.

## Data Availability

No applicable.
